# Antibiotic consumption patterns in older adults: a comparative study of people 65 years and older in and outside nursing homes, Belgium, 2016 to 2022

**DOI:** 10.2807/1560-7917.ES.2024.29.46.2400148

**Published:** 2024-11-14

**Authors:** Moira Kelly, Marc de Falleur, El Maati Allaoui, Laura Bonacini, Boudewijn Catry, Katrien Latour, Lucy Catteau

**Affiliations:** 1 Department of Epidemiology and public health, Sciensano, Brussels, Belgium; 2 National Institute for Health and Disability Insurance, Brussels, Belgium; 3 InterMutualistic Agency, Brussels, Belgium; 4 Faculty of Medicine, Université libre de Bruxelles (ULB), Brussels, Belgium; 5 Faculty of Medicine and Pharmacy, Université de Mons (UMons), Mons, Belgium

**Keywords:** Antimicrobial consumption, antimicrobial resistance, elderly, older patients

## Abstract

**Background:**

Inappropriate antimicrobial consumption (AMC) drives the emergence of antimicrobial resistance. Institutionalised, older populations are associated with antimicrobial treatments of longer duration and broader spectrum than recommended, higher rates of multidrug-resistant infections and poorer outcomes for resistant infections. Yet systematic, national monitoring of AMC in nursing home (NH) residents is lacking.

**Aim:**

To perform a retrospective analysis of antibiotic consumption in Belgian NHs, we compared analogous populations inside and outside NHs. We aimed to provide a blueprint for establishing surveillance of NH AMC, based on national reimbursement data.

**Methods:**

The National Institute for Health and Disability Insurance supplied reimbursement AMC data for outpatients from 2016 to 2022. Data were classified by the Anatomical Therapeutic Chemical system, expressed as defined daily doses (DDD) and aggregated by prescription month, patient age, sex and residency inside/outside a NH. The number of ensured beneficiaries, aggregated by the same demographic variables, was collected from the Intermutualistic Agency. We compared the DDDs per 1,000 beneficiaries per day, along with secondary metrics for national and international targets for analogous populations inside and outside NHs.

**Results:**

Total antibiotic consumption decreased in both populations but remained twofold higher in NH residents. Proxy prescription quality metrics were consistently less favourable within NHs and diverged further during the COVID-19 pandemic. Distinct consumption patterns and greater seasonal fluctuations were observed in NH residents.

**Conclusion:**

Given the different infection risks and higher antibiotic consumption of NH residents, AMC surveillance and antimicrobial stewardship efforts targeting this fragile population are needed.

**Key public health message**

**What did you want to address in this study and why?**

Inappropriate antimicrobial consumption (AMC) drives antimicrobial resistance (AMR). Current systematic AMC surveillances often overlook nursing homes, despite evidence that residents may (i) more often receive treatments outside of clinical guidelines, (ii) be more sensitive to inappropriate treatment and (iii) experience higher rates of AMR. We therefore wanted to analyse antibiotic consumption in Belgian nursing homes.

**What have we learnt from this study?**

Consumption rates in nursing homes exceeded twice those of the population outside nursing homes when comparing the same age group and sex. Seasonal fluctuations and distinct patterns in the types of antibiotics consumed were more pronounced inside nursing homes. Notably, consumption patterns were more divergent during the COVID-19 pandemic period from 2020 to 2022.

**What are the implications of your findings for public health?**

This study illustrated both a potential methodology for establishing a national-level surveillance of antibiotic consumption in nursing homes, and the need for such a system given the difference in consumption patterns between nursing home residents and analogous external populations.

## Introduction

Nursing homes (NHs) are an important blind spot in current antimicrobial consumption (AMC) surveillance efforts that aim to slow the emergence of antimicrobial resistance (AMR). Appropriate antimicrobial therapy varies according to population and clinical setting, making sector-specific AMC surveillance and guidelines crucial [1,2].

Belgium has a growing older population, with 2.3 million of its 11.5 million residents in 2021 older than 65 years. With one of the highest rates of NH beds per 1,000 citizens in the European Union/European Economic Area [3] and high bed occupancy rates (95–98%) [4], roughly 5% of those aged 65 and over reside in NHs. Frequent prescription of antimicrobials for NH residents, including over-prescribing broad-spectrum agents, prescribing antimicrobials for cases where such treatments are contraindicated, or prescribing unjustified prolonged treatment, raises concerns about inappropriate antimicrobial use [5-7]. Elderly patients may be more likely to suffer from comorbidities, and particularly patients with dementia may be unable to express or describe clinical symptoms. This can complicate diagnosis and, together with increased susceptibility to serious sequelae, may increase antimicrobial prescribing among practitioners. Antimicrobial treatment itself carries potential side effects such as *Clostridioides difficile* infection or impaired muscular, kidney or liver function, with 20% of adverse drug events in NHs attributed to antimicrobial therapies [8]. Furthermore, the overuse of antimicrobials in NHs may create reservoirs of multidrug-resistant organisms (MDROs) [9]. Because this frail population has both increased rates of morbidity and mortality in the face of MDRO infections [10], along with higher rates of hospitalisation and care interactions, there is an increased potential for introducing such difficult-to-treat organisms between and within care settings.

Despite the clear importance of appropriate antimicrobial use in NHs, considerable challenges have hindered local or national AMC surveillance programmes in these settings. Belgian national AMC surveillance primarily relies upon the Pharmanet reimbursement database [1,11-13], covering ca 99% of the Belgian population. In contrast to hospital prescriptions, which are uniformly managed through a single registry system and dispensed via on-site pharmacies, NH residents typically retain their primary general practitioner (GP), with community-based pharmacies delivering prescriptions. Consequently, distinguishing prescriptions specifically intended for NH residents presents a logistical challenge. Furthermore, effective surveillance efforts require accurate data on population size and demography, but no unified national database for NH residency currently exists in Belgium. As a result, our understanding of NH AMC in Belgium has largely relied on voluntary point prevalence surveys [14,15]. Similar challenges are observed in many European countries, with AMC data for NHs often hidden within surveillance of consumption in the ambulatory sector, hospital sector or a mixture of both [1].

However, in 2015, the recording of reimbursements of prescriptions for NH residents in Belgium was changed, allowing these prescriptions to be identified. In Belgium, health insurance is obligatory for all residents older than 25 years, thus more than 99% of older patients are included in reimbursement databases. This shift enabled the first retrospective analysis of antibiotic consumption data in Belgian NHs, marking a notable improvement in our ability to comprehensively assess and understand antibiotic consumption in these settings.

## Methods

We performed a retrospective study of antibiotic prescription deliveries to patients inside and outside NHs between 2016 and 2022 in Belgium, using the latest available reimbursement data from the Pharmanet database [13].

### Prescription data

Reimbursement data for antibiotic prescriptions delivered by community pharmacies, between January 2016 and August 2022, were provided by the National Institute for Health and Disability Insurance (NIHDI) [13] from the Pharmanet database. Pharmanet aggregates reimbursement data from the seven health insurance funds in Belgium, covering 99% of the Belgian population in 2021. Damian et al. found that Pharmanet reimbursement data accurately reflected total community antibiotic consumption, based on retail sales data across all classes except fluoroquinolones (due to changes in reimbursement criteria introduced in 2018 [12]) [16]. Antimicrobials were classified according to the Anatomical Therapeutic Chemical (ATC) classification of the World Health Organisation (WHO) [17], focusing on all medications belonging to the J01 group (antibacterials for systemic use).

Consumption was quantified in defined daily doses (DDDs, WHO version of January 2023) [17] aggregated by month and year of prescription, patient sex, age group, and residence inside or outside an NH. While the standard DDD may not be the appropriate dose for elderly patients in all cases, this unit allowed standardised comparison of consumption. Age groups (65–74 years, 75–84 years and ≥ 85 years) were defined by year of birth. Sex was collected as a binary variable from the patient’s health insurance information. Residency in an NH was based on a series of Pharmanet variables, primarily relying on a 2015 mandate that all tablet drugs delivered to NH residents be identifiable by either a specific delivery label or exception note, added by the pharmacist at the point of prescription delivery. Inaccurately labelled reimbursements may have been missed. The dataset excludes non-reimbursed treatments, imported products and magistral preparations (medications prepared by the pharmacist). Moreover, due to the extended potential reimbursement period (up to 2 years after prescription), the most recent, fully detailed annual period available for this study was 2021.

### Population data

Population data, stratified by the same demographic variables as the prescription data, were obtained from the Intermutualistic Agency (IMA, www.aim-ima.be). This dataset also compiles information from the seven health insurance funds in Belgium. The IMA-Atlas (https://ima.incijfers.be) includes the total count of ensured beneficiaries and provides estimates for the proportion of each population residing in NHs for age groups 65–74 years, 75–84 years and ≥ 85 years. The estimated proportional NH residency is determined from reimbursement data for residency between 28 March and 4 April each year as the numerator, with the total number of beneficiaries older than 65 years on 30 June in the IMA database who did not die between January and March as the denominator [18]. This methodology, developed and validated by a working group of Belgian experts from insurance funds, the NHDI, nursing care and nursing home care providers, is the standard approach used by the IMA. For this manuscript, the methodology was further validated using data from surveillance programmes within NHs and residency data for Flemish NHs.

For the comparable external population, the total number of beneficiaries, aggregated by sex and age group (65–74 years, 75–84 years and ≥ 85 years) was extracted from the IMA database, deducting the estimated number of beneficiaries residing in NHs per group. 

### Antibiotic consumption metrics

We compared the antibiotic consumption metrics for national and international targets for analogous populations inside and outside NHs.

#### Total J01 consumption

Total consumption was quantified as DDD per 1,000 beneficiaries per day (DBD), excluding antibiotic prescriptions delivered to patients younger than 65 years.

#### Secondary indicators for J01 prescription quality

We also considered secondary indicators for improving prescribing practices, such as encouraging fewer broad-spectrum antibiotic treatments and preserving newer generation antibiotics, namely:

- the percentage of total amoxicillin and amoxicillin-clavulanic acid consumption that comprises amoxicillin-only deliveries;
- the ratio of medications classified as second-line vs first-line treatments, according to the European Surveillance of Antimicrobial Consumption Network (ESAC-NET) [1], where first-line refers to penicillins with extended spectrum (J01CA), beta-lactamase-sensitive penicillins (J01CE), beta-lactamase-resistant penicillins (J01CF), first-generation cephalosporins (J01DB) and erythromycin (J01FA01), and second-line refers to amoxicillin-clavulanic acid (J01CR), second- and third-generation cephalosporins (J01DC and J01DD), fluoroquinolones (J01MA), and macrolides excluding erythromycin (J01FA except J01FA01);
- the proportions of medications classified by the WHO as Access, Watch or Reserve (AWaRe) [19], with ‘Access’ antibiotics comprising first-line and second-line therapies offering optimal therapeutic value while minimising the potential for resistance, ‘Watch’ consisting of antibiotics with assumed higher AMR potential, prioritised in stewardship and monitoring efforts, and ‘Reserve’ encompassing antibiotics of last resort, reserved for the treatment of confirmed or suspected infections caused by MDROs [20].

#### Urinary tract infection treatments

Information regarding the indication for antimicrobial treatment was unavailable. However, treatments for urinary tract infections (UTIs) are largely standardised, as outlined in the 2021 NIHDI medication use-UTI report, and national and European guidelines [20-22]. These include: ofloxacin (J01MA01), ciprofloxacin (J01MA02), norfloxacin (J01MA06), levofloxacin (J01MA12), nitrofurantoin (J01XE01), nifurtoinol (J01XE02), fosfomycin (J01XX01), co-trimoxazole (J01EE01) and trimethoprim (J01EA01). Reimbursement of these medications were therefore used as a proxy for treatments used to treat UTIs, which are the most common indication for antimicrobial treatment in Belgian NHs [14,15].

#### Analysis

All statistical analyses including Spearman’s rank correlation, Wilcoxon signed-rank tests and student t-tests were performed in R version 4.2.1 using base functions, with generalised linear models (GLMs) fit using the glm() function, data tidying and visualisation performed using the ‘tidyverse’ library [21]. Consumption data with missing patient age or sex details were removed before analysis (this amounted to less than 0.00042% of data by DDD).

## Results

Despite comprising roughly 5% of the beneficiaries over 65 years of age, residents of NHs accounted for more than 10% (range: 10.1–11.8 between 2016 and 2021) of outpatient J01 antibiotic consumption in this age group. The annual proportion of total J01 prescriptions delivered within NHs to patients under 65 years of age ranged from 2.9 to 3.4%. During the study period, the population over 65 years increased, while the proportion residing in NHs decreased. Supplementary Table S1 provides more details on the Belgian NH population.

### Total consumption of antibiotics for systemic use

**Figure 1:**
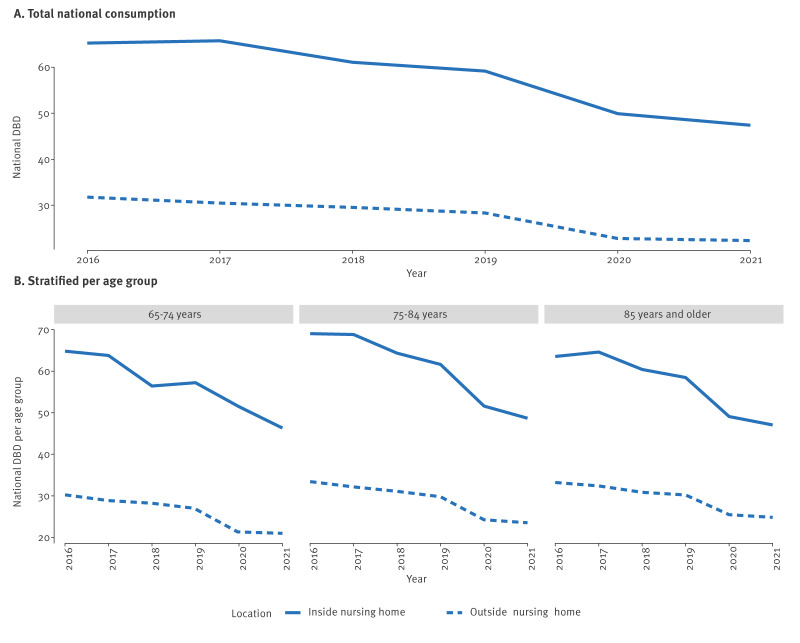
Total J01 antibiotic consumption expressed in defined daily doses per 1,000 ensured beneficiaries/day inside and outside nursing homes, Belgium, 2016–2021

The total consumption of J01 antibacterials for systemic use, expressed as DBD, decreased over the study period in populations residing both inside (Spearman’s correlation of consumption (DBD) modelled as a function of time, rho = −0.94, p = 0.017) and outside (Spearman’s rho = −1.00, p = 0.003) NHs. Nevertheless, antibiotic consumption was consistently higher inside NHs (Wilcoxon signed rank test of consumption (DBD) modelled as a function of residence, r = 0.899, p = 0.031) regardless of age group (Figure 1). Within NHs, residents aged 75–84 years consumed the most J01 antibiotics (GLM with structure consumption modelled as a function of year and age, indicates a significant negative quadratic relationship between age group and consumption (coefficient estimate = −3.06, p = 0.008, McFadden’s pseudo R^2^ = 0.92)). Conversely, populations outside NHs exhibit a linear relationship (coefficient estimate = 2.41, p = 3.93 × 10^−4^, McFadden’s pseudo R^2^ = 0.92), with older patients consuming more antibiotics. In Supplementary Figure S1, we additionally provide GLM fit, coefficient estimates and confidence intervals.

DBD: defined daily doses/1,000 beneficiaries/day.

Source: Pharmanet [13]. Population (year) = 2,154,955 (2016); 2,190,923 (2017); 2,223,342 (2018); 2,263,717 (2019); 2,304,313 (2020); 2,329,016 (2021).

### Secondary indicators

**Figure 2:**
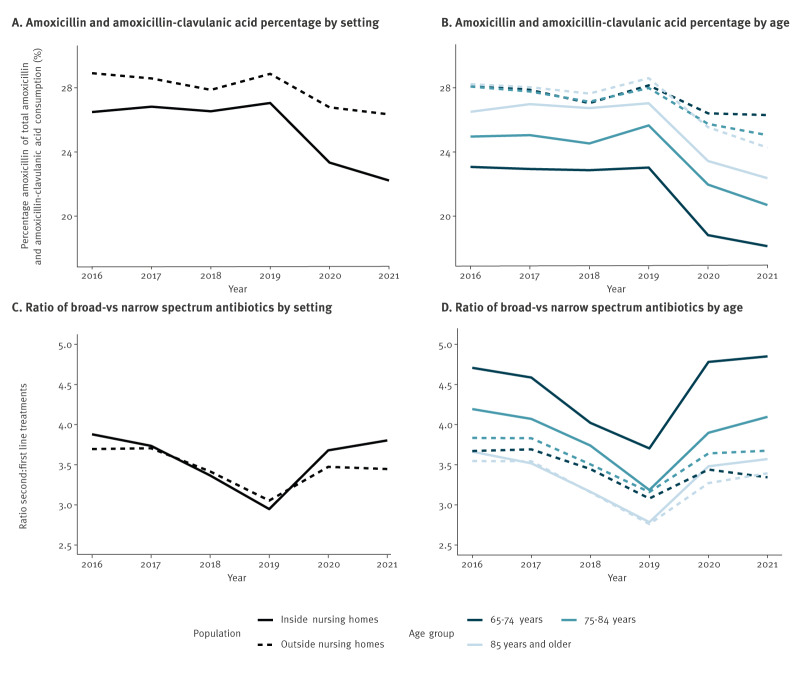
Secondary indicators for J01 consumption in nursing homes and outside, stratified by age groups, Belgium, 2016–2021

We observed relatively poorer J01 secondary indicator metrics within NHs compared with outside (Figure 2). Particularly when considering patient age, this was characterised by lower proportional consumption of amoxicillin (paired student t*-*test of amoxicillin:amoxicillin–clavulanic acid ratio modelled as a function of residence, t = −7.14, df = 17, p = 1.66 × 10^−6^) and higher proportional consumption of second-line treatments (paired student t-test of second-:first-line treatments ratio modelled as a function of residence*,* t *=* 4.073, df = 17, p = 0.00079). From 2017 to 2019 we observed a decrease in the ratio second-:first-line treatments. The proportion of total amoxicillin–amoxicillin clavulanic acid consumption representing amoxicillin remained stable during this period. By comparison, during the COVID-19 years 2020 and 2021, both metrics indicated increased relative prescription of broad-spectrum treatments in the populations older than 65 years, both within and outside NHs, that was particularly pronounced in younger (65–74 years) NH residents.

Panels A and B: percentage of total amoxicillin and amoxicillin–clavulanic acid consumption (defined daily doses) comprising amoxicillin-only deliveries. Panels B and D: ratio of the consumption of broad-spectrum penicillins, cephalosporins, macrolides (except erythromycin) and fluoroquinolones (J01(CR + DC + DD + (FA–FA01) + MA); second-line) to narrow-spectrum penicillins, cephalosporins and erythromycin (J01(CA + CE + CF + DB + FA01); first-line), as defined by the European Surveillance of Antimicrobial Consumption Network [1].

Source: Pharmanet [13]. Population (year) = 2,154,955 (2016); 2,190,923 (2017); 2,223,342 (2018); 2,263,717 (2019); 2,304,313 (2020); 2,329,016 (2021).

Considering consumption based on the AWaRe classification (Table), NH residents received higher proportions of both ‘Access’ and ‘Reserve’ group antibiotics compared with non-institutionalised individuals of the same age (coefficient = 0.03, p = 0.041, GLM with formula proportion of consumption (by DDD) modelled as a function of setting (inside vs outside NH) × WHO category). Conversely, lower proportions of ‘Watch’ list medications were found among NH residents (coefficient = −0.077, p = 6.8 × 10^−4^). For the detailed GLM coefficient estimates and confidence intervals we refer to Supplementary Figure S2.

**Table:** Percentage of World Health Organisation AWaRe-classified J01 antibiotics for the population ≥ 65 years inside and outside nursing homes, Belgium, 2016–2021

Category	Setting	2016	2017	2018	2019	2020	2021
Access	Inside nursing homes	64.7%	63.7%	66.9%	70.3%	70.4%	70.4%
	Outside nursing homes	60.6%	60.4%	64.2%	66.4%	66.6%	67.4%
Watch	Inside nursing homes	31.3%	32.3%	28.8%	25.2%	24.6%	23.9%
	Outside nursing homes	37.3%	37.5%	33.5%	31.3%	30.7%	29.7%
Reserve	Inside nursing homes	3.9%	4.0%	4.3%	4.5%	5.0%	5.7%
	Outside nursing homes	2.1%	2.2%	2.3%	2.3%	2.8%	2.9%

AWaRe: Access, Watch or Reserve.

### Anatomical therapeutic chemical third level

**Figure 3:**
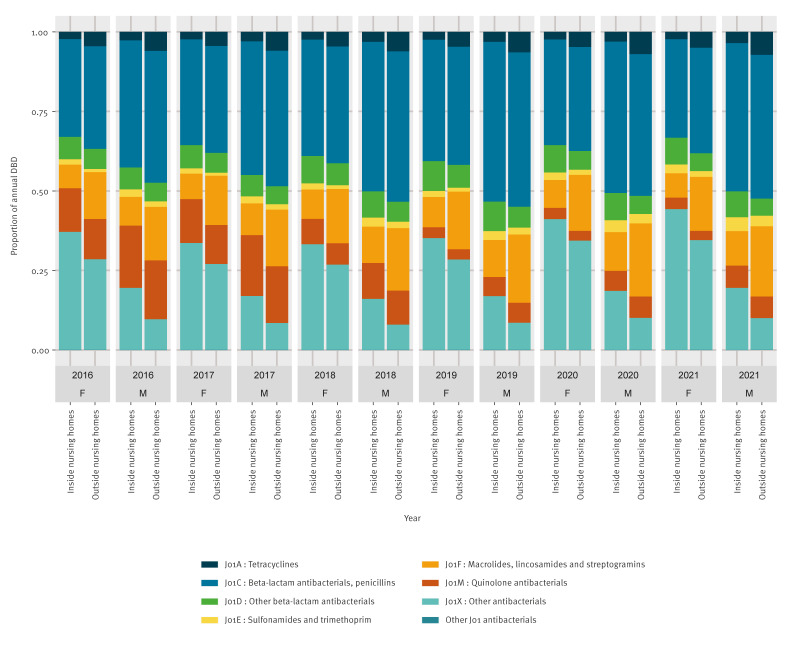
Consumption of J01 antibiotics^a^ inside and outside Belgian nursing homes, by sex, Belgium, 2016–2021

DBD: defined daily doses/1,000 beneficiaries/day; F: female, M: male.

^a ^By the World Health Organisation’s Anatomical Therapeutic Chemical third level.

The proportional consumption of J01X antibiotics, primarily used to treat UTIs, was notably higher in females, consistently higher in NH residents (both male and female), and increased over time (Figure 3). Supplementary Figure S3 provides more detailed comparison of the proportional consumption of antibiotics within the J01X class, by residency and sex. Conversely, quinolones (J01M) constituted a larger proportion of J01 consumption in males but varied little by residence inside or outside NHs. We found that tetracyclines (J01A) and macrolides (J01F) were consumed in greater volumes outside of NHs, regardless of sex.

Consumption as a proportion of total J01 annual DBD: n = 2,154,955 (2016); 2,190,923 (2017); 2,223,342 (2018); 2,263,717 (2019); 2,304,313 (2020); 2,329,016 (2021).

The proportion of ‘Other J01 antibacterials’ was too small to be visible in the graph (< 0.0006).

**Figure 4:**
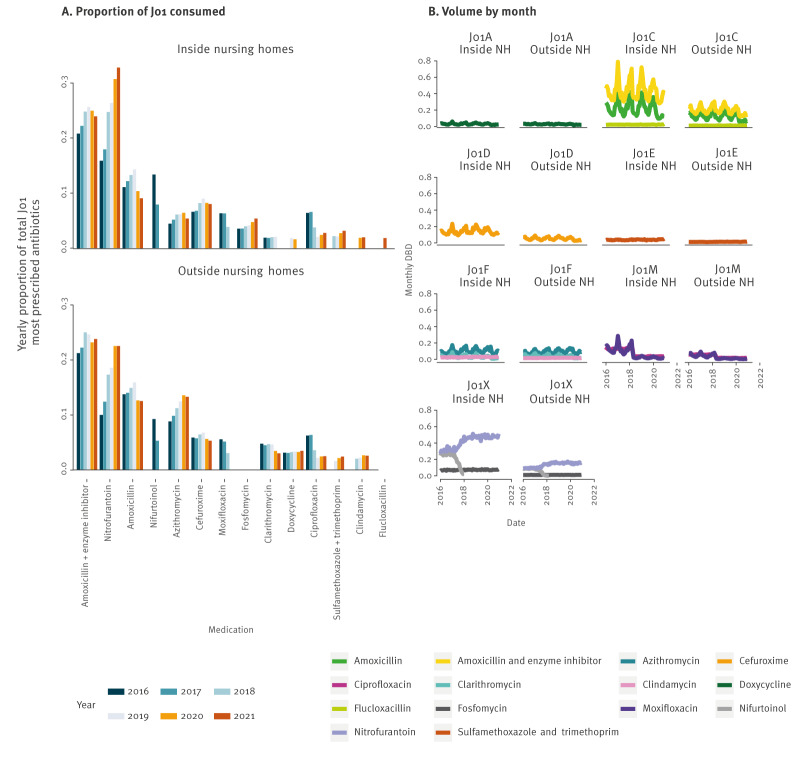
Top 10 most prescribed antibacterials for systemic use (J01) for people ≥ 65 years, outside and inside nursing homes, per year, Belgium, 2016–2021

DBD: DDD/1,000 beneficiaries/day; DDD: defined daily doses; NH: nursing home.

We observed a more concentrated antibiotic consumption profile inside NHs, with prescriptions for fewer different antibiotics delivered each year compared with the equivalent external population (mean number of products inside NHs = 33.5, outside NHs = 39, Wilcoxon signed rank test r = 0.899, p = 0.031). Furthermore, the annual top 10 most prescribed antibiotics (Figure 4) constituted a greater proportion of the total J01 prescribed volume (DDD) within NHs compared with the equivalent population outside NHs (mean proportion inside NHs = 0.925, outside NHs = 0.909, Wilcoxon signed rank test r = 0.899, p = 0.031). Across the study period, 14 different antibiotics were identified within the annual top 10 lists, with two compounds, fosfomycin and flucloxacillin, found exclusively in the NH most used products. Fosfomycin is primarily used to treat or prevent lower UTIs and appeared every year in the 10 most prescribed antibiotics in NHs, while flucloxacillin, a beta-lactam antibiotic primarily used to treat skin infections, appeared in the NH most used antibiotics in 2021 but not in other years or in the external population ≥ 65 years. While the J01X antibacterials showed only minor oscillations across the year, we observed notable winter peaks in consumption of the beta-lactam antibiotics (ATC groups J01C and J01D), as well as the J01F macrolides and J01M quinolones. As shown in the peak-to-trough ratios outlined in Supplementary Table S2, for the majority of the most commonly prescribed antibiotics, these seasonal fluctuations showed greater magnitude in NH populations than in the equivalent external population (Figure 4B).

Panel A shows the top 10 most prescribed J01 antibiotics each year, expressed as a proportion of total J01 consumed by that population that year (calculated in DDD), inside and outside nursing homes. Panel B shows the volume, expressed in DBD, delivered each month inside and outside nursing homes, for all antibiotics featured in the top 10 most prescribed antibiotics, grouped by third level Anatomical Therapeutic Chemical Classification [17].

Population (year) = 2,154,955 (2016); 2,190,923 (2017); 2,223,342 (2018); 2,263,717 (2019); 2,304,313 (2020); 2,329,016 (2021).

**Figure 5:**
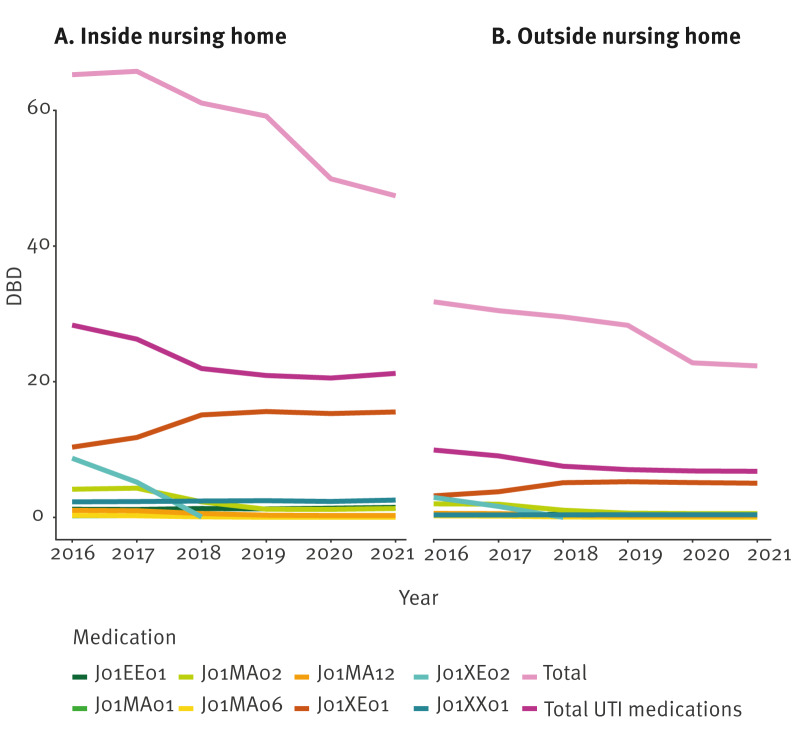
Evolution of prescription volumes of urinary tract infection medications (expressed in defined daily doses/1,000 beneficiaries/day) to individuals ≥ 65 years, inside and outside nursing homes, Belgium, 2016–2021

DBD: defined daily doses/1,000 beneficiaries/day; UTI: urinary tract infection.

Notably, four of the most used antibiotics in NHs (ciprofloxacin, nitrofurantoin, nifurtoinol and fosfomycin) were primarily prescribed for the treatment of UTIs. Treatments for UTI accounted for a substantial proportion (35.4–44.8%) of annual J01 antibiotic prescriptions within NHs during the study period, and UTI medications were disproportionately consumed by NH residents: while NH residents received on average 10.1–11.8% of all J01 prescriptions, 14.6–15.3% of UTI medication was consumed by NH residents. Supplementary Table S3 outlines the proportional consumption of UTI medications in the NH and external population. We found the Belgian consumption of such medications to be more resistant to efforts aimed at reducing antibiotic consumption. Unlike other antibiotic groups that showed notable reductions during the COVID-19 years 2020 and 2021, these specific antibiotic classes did not exhibit similar declines (Figure 5). We observed changes in the relative popularity of these medications, however, their overall consumption (DBD) remained stable from 2018 to 2021.

UTI medications considered here include: ofloxacine (J01MA01), ciprofloxacin (J01MA02), norfloxacin (J01MA06), levofloxacin (J01MA12), nitrofurantoin (J01XE01), nifurtoinol (J01XE02), fosfomycin (J01XX01), co-trimoxazole (J01EE01) and the total of these seven medications combined (total UTI medications). These volumes are compared with the volume of all J01 antibacterials (antibacterial for systemic use). Population (year) = 2,154,955 (2016); 2,190,923 (2017); 2,223,342 (2018); 2,263,717 (2019); 2,304,313 (2020); 2,329,016 (2021).

## Discussion

This longitudinal retrospective study of antibiotic consumption in Belgian NHs revealed higher antibiotic consumption within NHs compared with the corresponding non-institutionalised population. While NH residents in Belgium constitute ca 5% of the total health insurance beneficiaries over 65 years, they consume over 10% of J01 antibiotics in that age group. This finding is in line with observations in Scandinavian countries [5,23].

Total J01 consumption notably decreased between 2016 and 2021, both outside and within NHs. The greatest decrease was observed in the first two COVID-19 pandemic years 2020 and 2021, particularly within NHs, coinciding with notable increases in infection prevention and control measures [15], which may have reduced the spread of infections requiring antibiotic treatment. Conversely, primary care was reorganised during the pandemic, with restrictions potentially influencing the timely diagnosis and treatment of some conditions. This also coincided with increased efforts concerning antimicrobial stewardship and infection prevention and control in NHs in Belgium, e.g. through the implementation of hospital outbreak support teams aimed at disseminating hospital expertise into NHs [24].

We observed different distributions of antibiotic types consumed by NH residents vs the external population. While both populations showed seasonal fluctuations in the consumption of antibiotic groups such as beta-lactams, macrolides and quinolones, fluctuations were more pronounced in NH residents, perhaps linked to seasonal peaks in respiratory infections, which are more prevalent in NH settings [15,25]. Such peaks may be particularly notable given recent findings that increased seasonal resistance to all antibiotics positively correlates with winter peaks in penicillin and macrolide usage [26]. We observed notable reductions in total and seasonal consumption of J01M quinolones (with subsequent impacts on indicators such as the ratio second-:first-line treatments) after 2018 due to changes in the reimbursement criteria – with reimbursement restricted to a limited number of clinical scenarios [12]. We also observed different antibiotic consumption patterns when considering patient age. Within the external population, increasing age was associated with higher consumption, a finding in-line with other studies [27]. However, within nursing homes the middle age group (75–84 years) showed the highest consumption. This may be due to lower rates of infections in the highest age group, or perhaps lower rates of diagnosis of infectious morbidities due to difficulties expressing symptoms in the face of lower mobility and higher rates of dementia [28].

Prior studies have identified UTIs as the most common reason for antibiotic consumption within Belgian NHs [15], with a notable proportion of treatments classified as preventative or prophylactic [15,25], contrary to European guidelines [29]. This issue has also been the focus of regional public health campaigns [30] and is therefore of interest in the surveillance of AMC within Belgian NHs. Indeed, medications for UTIs accounted for the largest proportion of antibiotic consumption within NHs in our data. While we observed varying relative popularity of UTI medications, probably influenced by the market removal of nifurtoinol due to safety concerns in 2017 [22] and the reduction in fluoroquinolones reimbursement in 2018 [12], the overall consumption was constant. 

Since 2020, Belgium has had a national action plan in place to contain antimicrobial resistance (NAP-AMR) [2], including targets to reduce AMC in the ambulatory and hospital sectors. As NH AMC data are reported within outpatient data, we assessed the progress and contribution of NH antibiotic consumption to the NAP-AMR targets in that sector. While there has been progress towards a 40% total antibiotic consumption reduction, we observed a deterioration within both populations for secondary indicators, with NH residents showing a twofold poorer performance. Supplementary Table S4 details the progress towards these targets in the NH and external populations.

Our work represents the first retrospective analysis of antibiotic reimbursement data for the national NH community in Belgium. Two advantages of this methodology are (i) the possibility to present nationwide data (rather than a selected sample) and (ii) the absence of any additional work for NH staff, who particularly during the COVID-19 period had limited labour capacity to participate in additional surveillances. Furthermore, while no indication for prescription was provided, the compound and temporal detail of the dataset allowed indirect estimation of treatment for more specific conditions such as UTIs and the observation of seasonal variations in treatment prescriptions.

We must, however, also acknowledge certain assumptions and limitations. Primarily, estimates of NH antibiotic consumption are based on a combination of variables from the Pharmanet data and may not reflect consumption within NHs with 100% accuracy. Our dataset excludes non-reimbursed treatments and magistral preparations, leading to potential underrepresentation of certain treatments, particularly quinolones. We also only included antibiotic deliveries through community pharmacies, excluding a small fraction of NHs delivered by hospital pharmacies (1% or fewer of NH antibiotic consumption (in DDD)). Supplementary Table S5 details the consumption volume and number of nursing homes receiving prescriptions through hospital pharmacies. The 2-year reimbursement process also significantly delays compiling a complete dataset. Furthermore, the estimation of the NH population derived from reimbursement data, estimated on a single week per year, limits the recognition of fluctuations over time. Since 2019, NHs in Belgium have been under regional rather than federal jurisdiction, with regional variation in the availability of more detailed resident data. Particularly during the period 2019 to 2021, problems in the collection and centralisation of data may have led to an underestimation of the number of NH residents. Improving data on NH residencies, including length of stay, would greatly enhance our understanding of AMC within that sector.

Given the ageing Belgian population [31], improving antimicrobial stewardship in NHs becomes increasingly crucial to meet national and international AMC targets and enhance the health and welfare of this expanding, frail population.

## Conclusion

The shortcomings of currently available datasets, the elevated vulnerability of NH residents and their disproportionately high AMC, particularly of second-line treatments and WHO ‘Reserve’ antibiotics, underscore the need for targeted surveillance efforts and specific AMC targets for this population. An ideal system should enable timely collection and analysis of patient-level data, including prescription indication. Moving from aggregated datasets to individual- and institution-level data would enhance our understanding of localised usage patterns on a national scale and support targeted actions. Establishing such surveillance systems – ideally with local epidemiological diagnostic and antimicrobial susceptibility data – could support the development of effective benchmarking and feedback. 

## Supplementary Data

429000 Supplement
