# Supplementary material for: Antibiotic consumption patterns in older adults: a comparative study of people 65 years and older in and outside nursing homes, Belgium, 2016 to 2022
Source: Euro Surveill. 2024 Nov 14;29(46):2400148. doi: 10.2807/1560-7917.ES.2024.29.46.2400148 (PMC11565649; doi:10.2807/1560-7917.ES.2024.29.46.2400148)
Supplement: Supplement [file 24-00148_KELLY_Supplement.pdf]

SUPPLEMENTARY INFORMATION:

ANTIBIOTIC PATTERNS IN OLDER ADULTS : A COMPARATIVE STUDY OF 65+ POPULATIONS IN AND BEYOND BELGIAN NURSING HOMES, 2016-2022

This supplementary material is hosted by *Eurosurveillance* as supporting information alongside the article "ANTIBIOTIC PATTERNS IN OLDER ADULTS : A COMPARATIVE STUDY OF 65+ POPULATIONS IN AND BEYOND BELGIAN NURSING HOMES, 2016-2022 ", on behalf of the authors, who remain responsible for the accuracy and appropriateness of the content. The same standards for ethics, copyright, attributions and permissions as for the article apply. Supplements are not edited by *Eurosurveillance* and the journal is not responsible for the maintenance of any links or email addresses provided therein.

Supplementary Figures

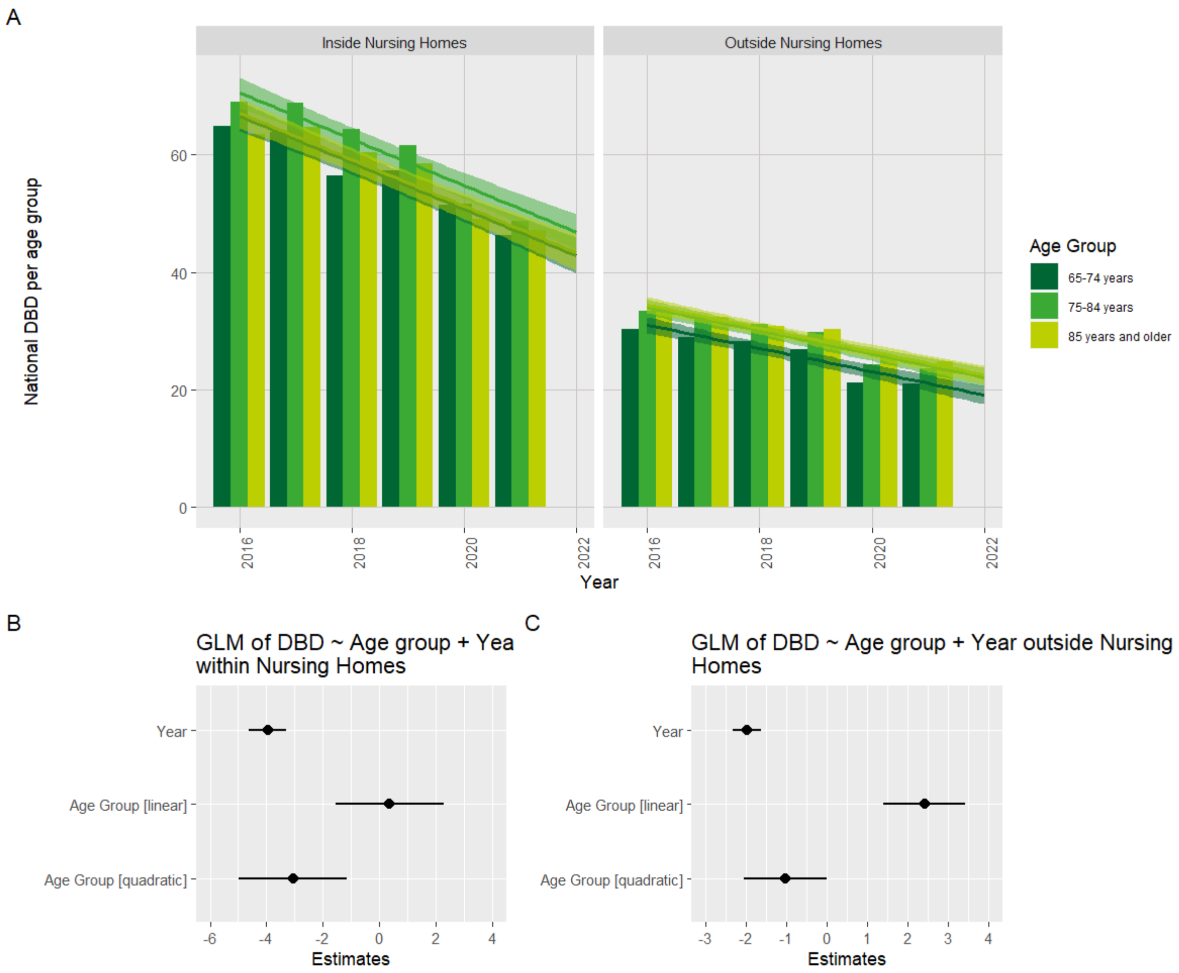

Supplementary figure 1. (A) Consumption of antibiotic agents in Belgian elderly expressed in DBD (Defined Daily Dose per 1000 beneficiaries per day) stratified by age group. Bars represent observed consumption data, predicted consumption (solid lines) with confidence intervals (shaded regions) of a generalised linear model (GLM) with structure consumption ~ year + age group, with age group treated as an ordinal categorical variable are also shown. Coefficient estimates of GLMs with structure consumption (DBD) ~ Age group ~ Years (2016-2022) are shown for consumption within nursing homes **(B)** and outside of nursing homes **(C)**.

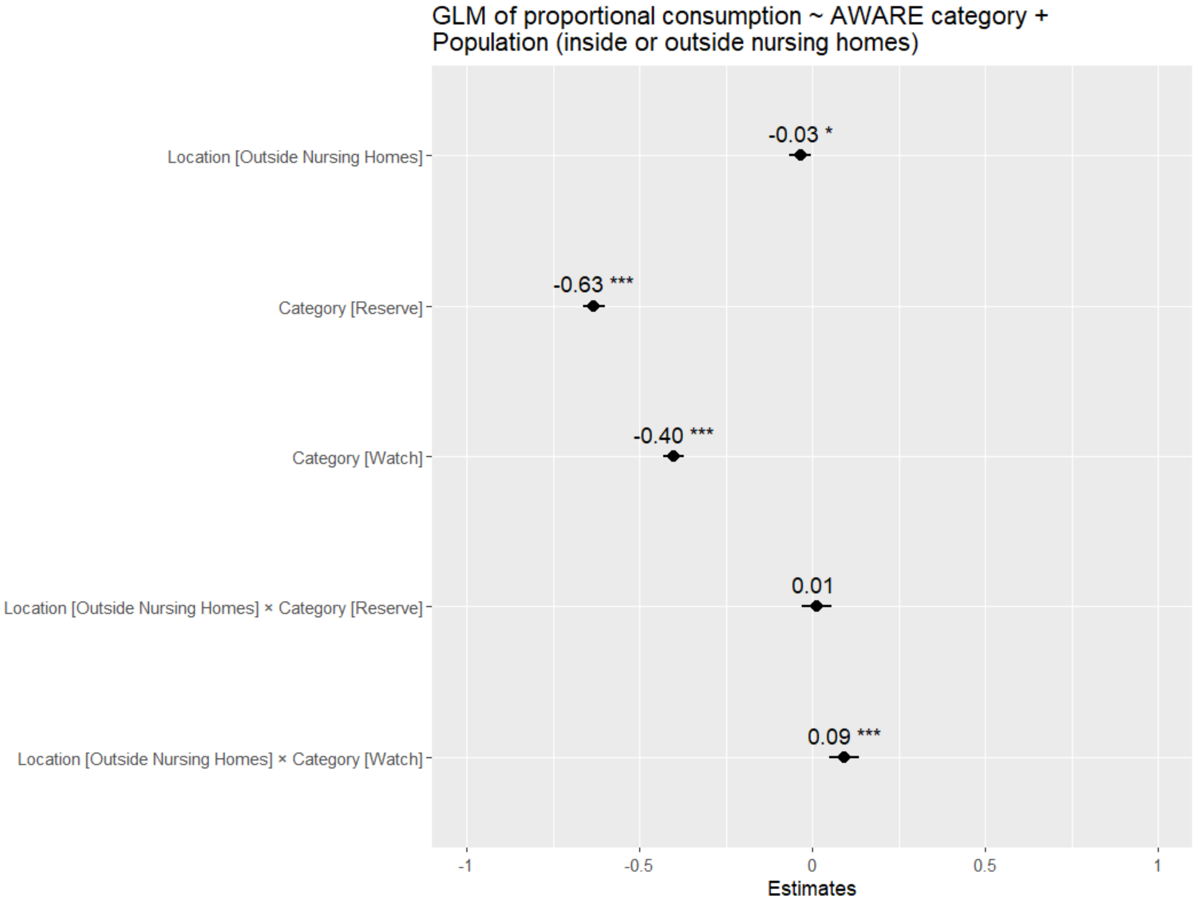

Supplementary figure 2. Model estimates for Generalised Linear Model (GLM) with structure proportion of total consumption ~ AWARE category * Residence inside or outside a Belgian nursing home (2016-2022). Numerical values depict the value of the estimate of the coefficient, with the level of significance indicated by * = $p < 0.05$, ** = $p < 0.01$, *** = $p < 0.001$.

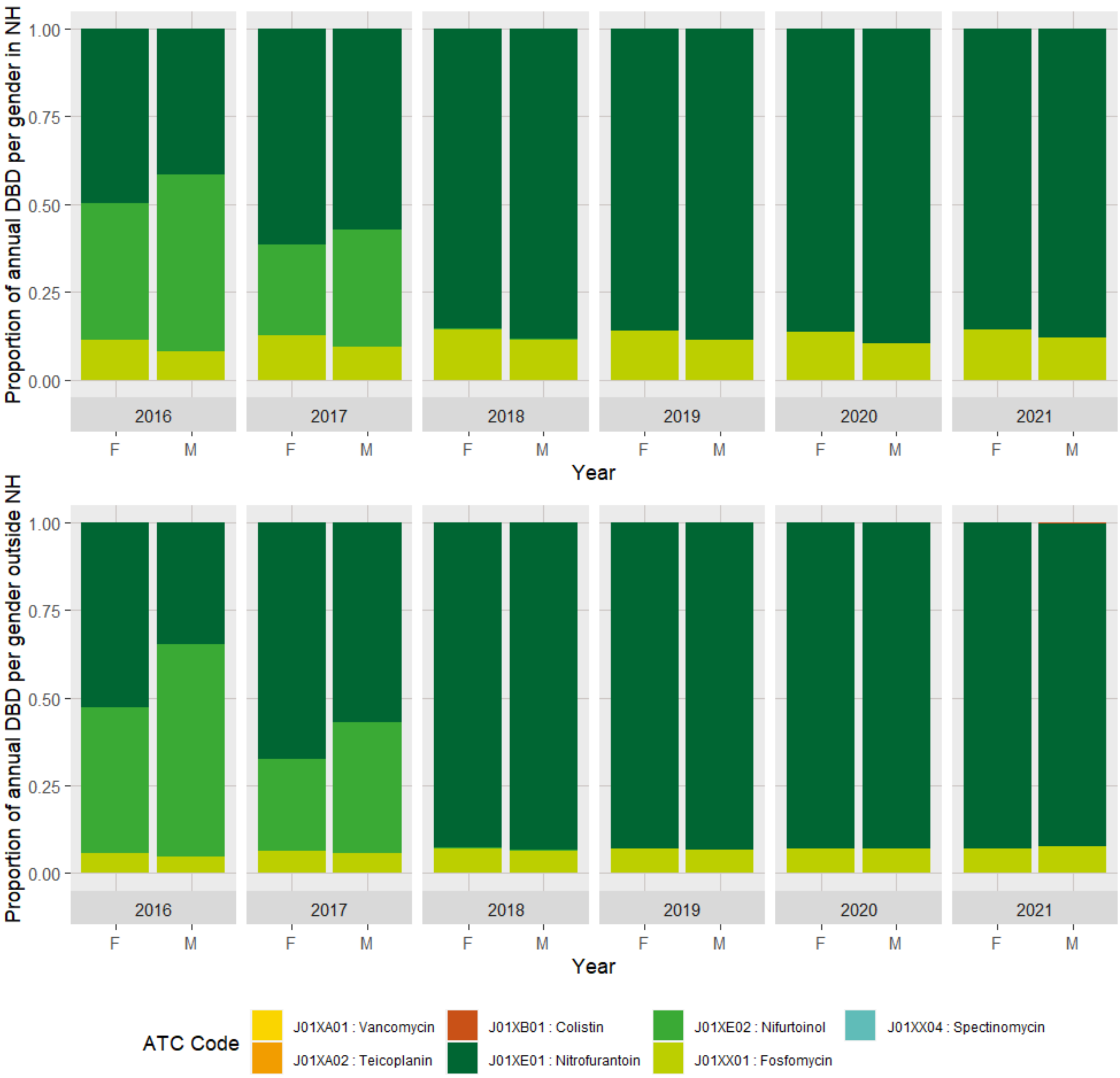

Supplementary Figure 3. Bar charts indicating the proportional contribution of molecules within the J01X "other antibacterials" class to total consumption of J01X antibiotics each year in male and female residents inside and outside nursing homes (NH), Belgium, 2016-2021. F= female, M = male. DID = defined daily dose (DDD) per 1000 beneficiaries per day (DBD).

We observe that within the J01X "other antibacterials" class, which are largely used to treat urinary tract infections, we see greater proportional use of fosfomycin within nursing home (NH) residents, particularly female NH residents. Before being removed from the Belgian market in 2018, nifurtoinol was more popular for use in male patients. Following its removal from the market, the nifurtoinol proportion of J01X antibiotics has been converted to nitrofurantoin (another nitrofuran-derivative) use.

Supplementary Tables

Supplementary Table 1. Distribution of Belgians aged over 65 years residing inside or outside nursing homes stratified by age, sex and region, 2016-2021. Pop = population (N, x10^3), % = percentage (within subsection annually)

			Within Nursing Homes						Outside Nursing Homes					
			2016	2017	2018	2019	2020	2021	2016	2017	2018	2019	2020	2021
	Total	Pop	127	128	128	124	124	117	2,023	2,058	2,090	2,133	2,176	2,207
Age groups (years)	65-74	Pop	10	11	11	10	11	11	1,075	1,108	1,132	1,156	1,183	1,191
		%	8.1	8.4	8.6	8.3	8.5	9.3	53.2	53.9	54.2	54.2	54.4	54.0
	75-84	Pop	34	33	33	31	30	29	681	673	675	684	694	715
		%	27.1	26.0	25.3	24.7	24.6	24.6	33.7	32.7	32.3	32.1	31.9	32.4
	85+	Pop	82	84	85	83	83	78	267	277	283	293	299	301
		%	64.9	65.6	66.1	67.0	67.0	66.1	13.2	13.5	13.5	13.7	13.8	13.7
Region	Brussels	Pop	12	12	12	08	08	07	145	146	147	152	152	152
		%	9.7	9.5	9.5	6.5	6.4	5.9	7.2	7.1	7.0	7.1	7.0	6.9
	Flanders	Pop	71	73	73	76	76	73	1,249	1,272	1,294	1,317	1,345	1,366
		%	56.3	56.9	57.1	61.3	61.1	62.5	61.8	61.8	61.9	61.7	61.8	61.9
	Wallonia	Pop	43	43	43	40	40	37	628	640	650	665	679	688
		%	34.0	33.6	33.4	32.2	32.4	31.6	31.1	31.1	31.1	31.2	31.2	31.2
Sex	Male	Pop	31	32	32	32	32	31	909	928	946	967	990	1,005
		%	24.6	24.8	25.1	25.7	26.0	26.0	45.0	45.1	45.3	45.3	45.5	45.6
	Female	Pop	96	96	96	92	92	87	1,114	1,130	1,144	1,166	1,186	1,202
		%	75.4	75.2	74.9	74.3	74.0	74.0	55.1	54.9	54.8	54.7	54.5	54.5

Table 1 provides data on the population aged over 65 years in Belgium, distinguishing between those residing in NHs and those living independently. Between 2016 and 2021, the total population of individuals over 65 steadily increased. During the same period, the proportion of those residing in NHs decreased. NH residents are predominantly in the oldest age group, with 64.9% to 67.0% of the NH population being over 85 years of age. Flanders houses the majority of Belgian NH residents; accounting for 56.3 to 62.5% of the NH population between 2016 and 2021. It is worth noting that this region is also characterized by the highest concentration of older individuals, as evidenced by the majority of the population over 65 years of age residing there. Additionally, among Belgians aged 65 and above, women constitute 55% of the population (Supplementary Table 1). This sex disparity is inflated in NHs, where women represent a significant majority ranging from 74.0 to 75.4 %.

Since 2019, nursing homes in Belgium fall under regional rather than federal jurisdiction, with regional variation in the availability of more detailed resident data. Particularly during the early stages (years 2019-2021) of this transition there were some problems in collection and centralisation of resident data, such that estimates of the number of residents in this period may be incomplete and therefore an underestimation. It is worth noting that studies of NH residency in Belgium report a constant occupancy rate exceeding 95%[1]. Additionally, while this dataset focuses exclusively on residents over 65 years of age, regional data suggests that the population under 65 years of age constitutes a minimal proportion of NH residents (< 2.5%, data from Flemish government, personal communication). However, the COVID-19 era undeniably caused notable disruptions and changes in residency numbers, particularly impacting our study period.

Supplementary Table 2. Magnitude of seasonal variation (annual peak-to-trough ratios) of antibiotics included in top 10 products with greatest delivery volumes each year to patients over 65 years inside and outside nursing homes.

	Max seasonal variation (ratio max/min)				
	Inside NH				
Antibiotic	2016	2017	2018	2019	2020
J01AA02 : Doxycycline	1.83	2.80	2.32	2.16	2.29
J01CA04 : Amoxicillin	2.53	4.19	3.31	2.92	3.76
J01CF05 : Flucloxacillin	1.20	1.24	1.30	1.23	1.63
J01CR02 : Amoxicillin and enzyme inhibitor	1.76	2.61	2.15	2.35	2.04
J01DC02 : Cefuroxime	1.67	2.52	1.75	1.93	1.94
J01EE01 : Sulfamethoxazole and trimethoprim	1.28	1.30	1.31	1.53	1.52
J01FA09 : Clarithromycin	2.92	4.81	3.34	3.55	5.54
J01FA10 : Azithromycin	1.91	2.58	2.05	2.32	2.54
J01FF01 : Clindamycin	1.30	1.31	1.28	1.26	1.31
J01MA02 : Ciprofloxacin	1.18	1.18	4.15	1.39	1.25
J01MA14 : Moxifloxacin	2.13	4.67	16.21	3.16	3.95
J01XE01 : Nitrofurantoin	1.32	1.44	1.20	1.20	1.12
J01XE02 : Nifurtoinol	1.13	11.49	1.00		
J01XX01 : Fosfomycin	1.25	1.20	1.24	1.18	1.19
	Outside NH				
Antibiotic	2016	2017	2018	2019	2020
J01AA02 : Doxycycline	1.43	1.71	1.66	1.55	1.97
J01CA04 : Amoxicillin	2.34	2.83	2.79	2.56	3.41
J01CF05 : Flucloxacillin	1.34	1.31	1.46	1.45	1.37
J01CR02 : Amoxicillin and enzyme inhibitor	1.74	1.94	1.89	2.06	2.11
J01DC02 : Cefuroxime	2.13	2.90	2.23	2.36	2.86
J01EE01 : Sulfamethoxazole and trimethoprim	1.20	1.31	1.40	1.33	1.48
J01FA09 : Clarithromycin	2.40	3.28	3.18	3.03	4.21
J01FA10 : Azithromycin	2.00	2.10	2.01	1.86	2.05
J01FF01 : Clindamycin	1.17	1.14	1.23	1.17	1.30
J01MA02 : Ciprofloxacin	1.11	1.14	3.50	1.20	1.26
J01MA14 : Moxifloxacin	2.66	3.86	15.88	2.94	4.93
J01XE01 : Nitrofurantoin	1.13	1.62	1.29	1.19	1.18
J01XE02 : Nifurtoinol	1.09	24.55	91.31		
J01XX01 : Fosfomycin	1.14	1.21	1.28	1.18	1.25

Supplementary Table 3. Consumption of selected molecules commonly used to treat urinary tract infections in Belgian elderly. Proportion of total J01 antibiotic consumption each year (calculated as a percentage of consumption in Defined Daily Doses) in the population over 65 years residing inside or outside nursing homes (NH).

		2016	2017	2018	2019	2020	2021
Consumption as a % of all J01 within NH in >65yrs	J01MA01 : Ofloxacin	0.4%	0.4%	0.2%	0.1%	0.1%	0.1%
	J01MA02 : Ciprofloxacin	6.4%	6.6%	3.8%	2.0%	2.4%	2.8%
	J01MA06 : Norfloxacin	0.5%	0.4%	0.1%	0.0%	0.0%	0.0%
	J01MA12 : Levofloxacin	1.6%	1.5%	0.9%	0.6%	0.6%	0.6%
	J01XE01 : Nitrofurantoin	15.9%	17.9%	24.7%	26.4%	30.7%	32.8%
	J01XE02 : Nifurtoinol	13.4%	7.9%	0.1%	-	-	-
	J01XX01 : Fosfomycin	3.5%	3.6%	4.0%	4.1%	4.7%	5.4%
	J01EA01 : Trimethoprim*	0.0%	0.0%	0.0%	0.0%	0.0%	0.0%
	J01EE01 : sulphamethaxole + trimethoprim	1.9%	1.8%	2.2%	2.1%	2.7%	3.1%
	Total	43.4%	40.0%	35.9%	35.4%	41.2%	44.8%
% of total consumption in entire >65yrs population consumed inside NH	J01MA01 : Ofloxacin	6.9%	7.4%	7.1%	5.9%	7.1%	5.9%
	J01MA02 : Ciprofloxacin	11.6%	12.1%	11.7%	10.1%	10.7%	11.0%
	J01MA06 : Norfloxacin	7.7%	7.4%	6.9%	6.5%	5.4%	7.3%
	J01MA12 : Levofloxacin	9.9%	9.7%	9.3%	7.6%	6.9%	6.4%
	J01XE01 : Nitrofurantoin	17.0%	16.2%	15.3%	14.7%	14.5%	14.1%
	J01XE02 : Nifurtoinol	15.7%	16.6%	34.1%	0.0%	0.0%	0.0%
	J01XX01 : Fosfomycin	28.5%	28.4%	27.8%	26.7%	25.8%	25.7%
	J01EA01 : Trimethoprim*	0.0%	0.0%	0.0%	0.0%	0.0%	0.0%
	J01EE01 : sulphamethaxole + trimethoprim	15.4%	15.6%	15.1%	13.5%	13.4%	12.7%
	Total	15.2%	15.3%	15.2%	14.7%	14.6%	14.2%

*Trimethoprim delivered as a magisterial preparation is not included in our dataset. However, Belgian records from the European Healthcare-associated infections in long-term care facilities (HALT) survey suggest that trimethoprim is rarely prescribed in this setting.

Supplementary Table 4. Metrics included in the Belgian National Action Plan in the fight against antibiotic resistance, comparing performance inside and outside nursing homes (NH). Source: Pharmanet. DBD = Defined Daily Dose (DDD) per 1000 insured Beneficiaries per day. Ratio of second:first line treatments is the ratio of the consumption of broad-spectrum penicillins, cephalosporins, macrolides (except erythromycin) and fluoroquinolones (J01(CR+DC+DD+(FA–FA01)+MA); second-line) to narrow-spectrum penicillins, cephalosporins and erythromycin (J01(CA+CE+CF+DB+FA01); first-line), as defined by the European Surveillance of Antimicrobial Consumption Network, for inside and outside nursing homes (C) and stratified by age (D). Source = Pharmanet

Metric (Unit)	Population	2019	2021	Progress	Target for 2024
Total Consumption (DBD)	Inside NH	59.2	47.4	19.9% reduction	40% reduction
	Outside NH	28.3	22.3	21.2% reduction	
Amoxicillin/ (Amoxicillin+amoxicillin - clavulanic acid) (%)	Inside NH	35.8	27.5	23.2% reduction	increase to 80%
	Outside NH	39.2	34.5	12.0% reduction	
Ratio second : first line antibiotics	Inside NH	2.95	3.80	28.8% increase	consistent reductions
	Outside NH	3.05	3.45	13.1% increase	

Since 2020, Belgium has implemented the Belgian national action plan to combat antimicrobial resistance (NAP-AMR)[2], which includes targets to reduce AMC in the ambulatory and hospital sectors. As nursing home AMC data has always been included in the ambulatory sector reporting, it is important to assess the AMC metrics within nursing homes in comparison to the NAP-AMR targets in that sector. The first objective is to achieve a 40% reduction in total AMC in 2024 compared to 2019. Encouragingly, we observed 19.9% and 21.2% reductions in total AMC for the populations over 65 years of age, inside and outside NHs respectively, between 2019 and 2021. The second national target aims to reduce quinolones consumption to a maximum of 5% of total J01 AMC, However, due to changes in Belgian reimbursement criteria for quinolones in 2018[3], our dataset, which relies solely on reimbursement data from NIHDI, only includes a fraction of quinolone consumption. As a result, this objective was not assessed. The final two NAP-AMR targets pertain to prescription quality, aiming to decrease the proportion of broad-spectrum antibiotics out of the total J01 antibiotic consumption and achieve a target for amoxicillin consumption comprising 80% of total amoxicillin and amoxicillin-clavulanic acid consumption. However, both residents within NHs and individuals outside show no consistent reduction in second:first line treatments and are still far from this 80% amoxicillin national target. Notably, during the COVID-19 era, we observed a deterioration in these metrics, particularly within NHs. This pattern of increasing proportional broad spectrum antibiotic use during the COVID-19 era has also been observed in other countries [4, p. 19]. These trends may reflect an increase in AMR in NH populations during this period, as observed in COVID-19 patients in other clinical settings[5], and thus a requirement for broader spectrum antibiotic use. Alternatively, other factors such as the adoption of teleconsultations, an abundance of caution due to a lack-of-knowledge, or increased concern about the elevated risks of COVD-19 when associated with comorbidities during an epidemic may have influenced antibiotic selection.

Supplementary Table 5. Number of Belgian nursing homes with antibiotics for systemic use (J01) delivered through hospital associated pharmacies – and therefore not included in our analysis, along with their antibiotic for system use (J01) consumption (expressed in Defined Daily Doses (DDD)). Source: BeH-SAC database[6], Sciensano

	2016	2017	2018	2019	2020	2021
Number of Nursing homes with J01 prescriptions fulfilled by hospital pharmacies (excluded from database)*	24	22	22	21	22	21
Total J01 consumption per year in nursing homes delivered through hospital pharmacies (thousand DDD)	29.8	32.1	27.5	22.7	17.5	15.4
Total J01 consumption per year in nursing homes delivered through community pharmacies (thousand DDD)	3,021.1	3,067.9	2,857.4	2,679.1	2,253.6	2,029.7

[1] S. Dequeker *et al.*, "COVID-19 Clusters in Belgian Nursing Homes: Impact of Facility Characteristics and Vaccination on Cluster Occurrence, Duration and Severity," *Viruses*, vol. 15, no. 1, p. 232, Jan. 2023, doi: 10.3390/v15010232.

[2] FPS Health, Food Chain Safety and Environment, "Belgian 'One Health' National Action Plan on the fight against Antimicrobial Resistance (AMR) 2020-2024." Accessed: Nov. 10, 2022. [Online]. Available: https://www.health.belgium.be/sites/default/files/uploads/fields/fpshealth_theme_file/en-amr_one_health_national_plan_final_1.pdf

[3] H. Vermeulen, S. Coenen, N. Hens, and R. Bruyndonckx, "Impact of changing reimbursement criteria on the use of fluoroquinolones in Belgium," *J. Antimicrob. Chemother.*, vol. 76, no. 10, pp. 2725–2732, Oct. 2021, doi: 10.1093/jac/dkab255.

[4] X. Zhong *et al.*, "Impact of COVID-19 on broad-spectrum antibiotic prescribing for common infections in primary care in England: a time-series analyses using OpenSAFELY and effects of predictors including deprivation," *Lancet Reg. Health – Eur.*, vol. 0, no. 0, May 2023, doi: 10.1016/j.lanepe.2023.100653.

[5] B. J. Langford *et al.*, "Antimicrobial resistance in patients with COVID-19: a systematic review and meta-analysis," *Lancet Microbe*, vol. 4, no. 3, pp. e179–e191, Mar. 2023, doi: 10.1016/S2666-5247(22)00355-X.

[6] "Antimicrobiële consumptie in België: resultaten van de ESAC-Net en BeH-SaC monitoring - NOSO INFO." Accessed: Oct. 16, 2023. [Online]. Available: https://www.nosoinfo.be/nosoinfos/antimicrobiele-consumptie-in-belgie-resultaten-van-de-esac-net-en-beh-sac-monitoring/?lang=nl
